# Impact of anthropogenic and natural environmental changes on *Echinococcus* transmission in Ningxia Hui Autonomous Region, the People’s Republic of China

**DOI:** 10.1186/1756-3305-5-146

**Published:** 2012-07-24

**Authors:** Yu Rong Yang, Archie C A Clements, Darren J Gray, Jo-An M Atkinson, Gail M Williams, Tamsin S Barnes, Donald P McManus

**Affiliations:** 1 Molecular Parasitology Laboratory, Queensland Institute of Medical Research, Brisbane, Queensland, Australia; 2 Ningxia Medical University, Yinchuan, Ningxia Hui Autonomous Region, P. R. of China; 3 Griffith Health Institute, Griffith University, Brisbane, Queensland, Australia; 4 School of Population Health, University of Queensland, Brisbane, Queensland, Australia; 5 University of Queensland, Queensland Alliance for Agriculture and Food Innovation, Gatton, Queensland, Australia

**Keywords:** Ningxia Hui Autonomous Region of the Peoples Republic of China, *Echinococcus* transmission, Environmental changes, Intermediate host, Definitive host

## Abstract

*Echinococcus* transmission is known to be affected by various environmental factors, which may be modified by human influence or natural events including global warming. Considerable population growth in the last fifty years in Ningxia Hui Autonomous Region (NHAR), the People’s Republic of China (PRC), has led to dramatic increases in deforestation and modified agricultural practices. In turn, this has resulted in many changes in the habitats for the definitive and intermediate hosts of both *Echinococcus granulosus* and *E. multilocularis*, which have increased the risks for transmission of both parasites, affecting echinococcosis prevalence and human disease. Ecological environmental changes due to anthropogenic activities and natural events drive *Echinococcus* transmission and NHAR provides a notable example illustrating how human activity can impact on a parasitic infection of major public health significance. It is very important to continually monitor these environmental (including climatic) factors that drive the distribution of *Echinococcus* spp. and their impact on transmission to humans because such information is necessary to formulate reliable future public health policy for echinococcosis control programs and to prevent disease spread.

## Review

### Introduction

Globally, the zoonotic disease echinococcosis (hydatidosis) is one of the most important parasitic helminth diseases, with over three million people infected worldwide [1]. The two major species infecting man are *Echinococcus multilocularis*, causing alveolar echinococcosis (AE), and *Echinococcus granulosus*, the cause of cystic echinococcosis (CE). It is estimated that there are 0.38 million people with echinococcosis in China [2,3] and the disease burden in Disability-Adjusted Life-Years (DALYs) lost is greater there than any other country [4]. Of the 33 provinces/autonomous regions, 20 are considered to be endemic for *E. granulosus* and five for *E. multilocularis*[2,5]. Ningxia Hui Autonomous Region (NHAR) is hyper-endemic for both AE and CE in humans and animals, with some of the highest infection rates recorded anywhere [6]. Although there are many risk factors impacting upon the transmission dynamics of the *Echinococcus* species, the link between climate variables and disease risk from these parasites is increasingly being recognized [7]. Concern has been raised about the potential impact of climate change on the burden of echinococcosis [7-11], due to its direct influence on the *Echinococcus* free-living egg stage and its indirect influence on host species abundance and distribution through modification of food source availability and migration patterns. Additionally, the maintenance and persistence of the parasitic life-cycles requires stable predator-prey relationships that can be disrupted by environmental change [7,9,12].

Environmental factors impact on the density of intermediate hosts and affect egg survival, which is governed by ground temperature, moisture and precipitation. In the case of the small mammalian hosts of *E. multilocularis*, density is determined by the presence of certain vegetation types that provide suitable habitats, whereas for *E. granulosus* transmission, it is influenced by grassland availability for livestock [13]. Human echinococcosis is associated with poverty and poor hygiene practices, particularly in livestock-raising communities where the natural environment is suitable to maintain the life-cycle. Lack of adequate water and sanitation systems also contribute considerably to transmission as a result of contamination of the water supply with faeces from parasitised hosts [14]. NHAR is one of the poorest provinces in China [15], where a large number of people, particularly among the Hui Muslims, an ethnic minority comprising one third of the population [16], raise livestock. Previously published data of clinical records and the results of mass screening have shown that CE occurs throughout NHAR whereas AE occurs predominantly in a confluence area of three counties (Xiji, Guyuan and Haiyuan) [6]. Here we review the environmental (including climatic) factors that drive the distribution of *Echinococcus* spp. and their impact on human transmission in NHAR.

### Environmental change over time

**Figure 1:**
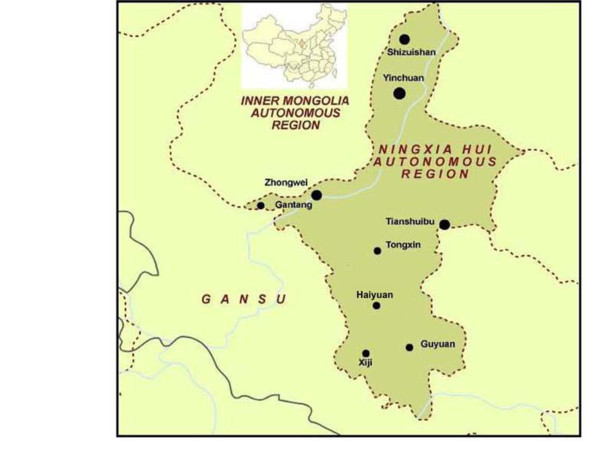
Location of Ningxia Hui Autonomous Region (NHAR) within China and its major cities and towns.

NHAR (Figure 1), located in Northwest China [17], has a continental climate with average temperatures that generally peak at 17–24°C in July and drop to -7–-10°C in January. Temperatures can, however, reach extremes of 39°C in summer and -30°C in winter. The diurnal temperature variation can exceed 17°C, especially in spring. Average annual rainfall varies from 190 to 700 mm, with more rain falling in the south of the region [18,19]. The natural geographic features divide NHAR into two main regions, northern and southern NHAR [20]. Although NHAR is sparsely settled, the vast plain of the Yellow River in the north has been irrigated for centuries and, over the years, an extensive system of canals has been built. Desert and grazing land make up most of the area except the northern fertile plain, which is ideal for growing crops and breeding livestock. In contrast to the northern parts, southern NHAR is mountainous and the people mainly raise livestock with limited farmland. No irrigation systems are present in the south and animal husbandry and agriculture are critically dependent on the environmental and climatic conditions prevailing throughout the year.

**Figure 2:**
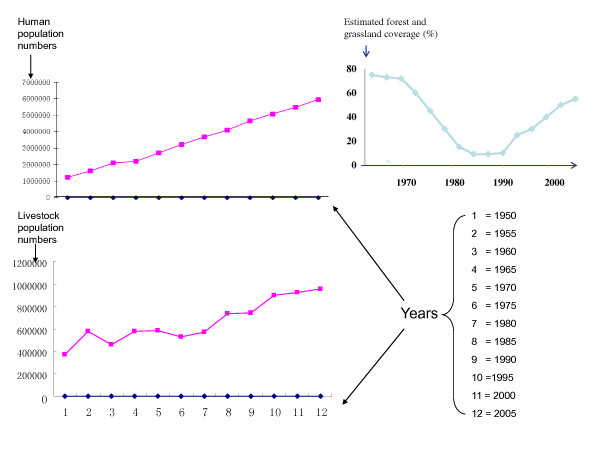
The increase in human (top left panel) and livestock population (bottom left panel) numbers in NHAR for the period 1950-2005, together with land use (forest and grassland coverage) changes over time (from the early 1970s to mid 2000).

Similar to the rest of China the population of NHAR has increased rapidly over the past 50 years (Figure 2), especially among the Hui community, resident in the south. This is due to the Chinese Government’s family planning policy, whereby a Chinese Han couple is permitted only one child whereas for ethnic minorities, two to three children are allowed. The marked population growth generally in NHAR has resulted in a substantial increase in the requirement for agricultural land, which has, in turn, resulted in extensive deforestation [16,17,21].

**Figure 3:**
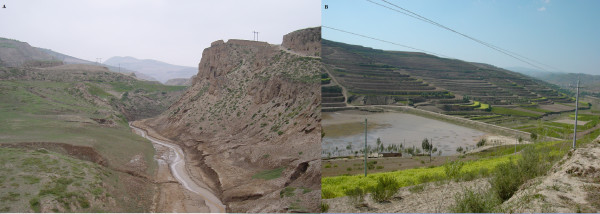
Photographs taken in southern NHAR in 2002-2003 showing erosion and sparse vegetation following deforestation and over-grazing by livestock (A); and creation of terraced agricultural farmland (B).

From the late 1970s to the early 2000s, NHAR lost large areas of original natural grassland due to urbanisation and the formation of cropland and woodland [8]. This was particularly the case in southern NHAR where land use changes (Figure 2) have resulted in the majority of livestock grazing land having been converted into terraced fields for production of wheat, potatoes, beans and alfalfa. This type of farmland has been extended to lower altitudes during the past five decades [8]. There is little or no natural vegetation left in the southern parts of NHAR following these periods of deforestation and a move towards more intensive agricultural production (Figure 3) [13]. At higher elevations of the Liupan Mountain range, which extends southward from NHAR across the eastern panhandle of Gansu province and into western Shaanxi province, pasture land was predominant several decades ago [8]. Currently, much of the remaining forest on southern Liupan Mountain (2100–2800 m) consists of secondary forest, although some patches of pristine forest can still be found [9]. Local government records indicate that, during the late 1970s, the valleys and lower slopes of southern NHAR were generally used for agricultural cropland while the upper slopes and hill tops were reserved for grazing [8,22]. This natural landscape has been modified into an artificial landscape comprising farmland and urban areas (Figure 2). Wildlife biodiversity has been affected by increasing soil erosion, changing local climatic conditions, such as more frequent droughts, floods and seasonal sand storms, and an increase in pest species. This is most pronounced in the mountainous areas of southern NHAR, where there used to be substantial wildlife habitats, and an abundance of foxes, wolves, dogs and wild large and small herbivorous mammals.

**Figure 4:**
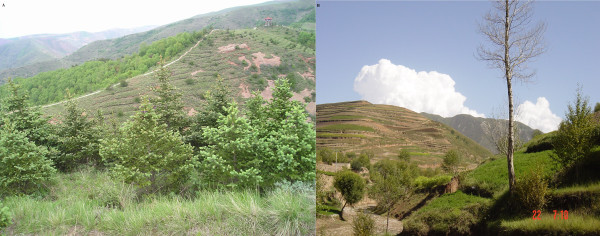
Photographs taken in southern NHAR in 2009-2011 showing reforestation of both grazing land (A) and previously terraced farmland (B).

The Chinese government is currently attempting to reforest eroded landscapes (Figure 4) and therefore sheep grazing has been reduced over the majority of NHAR, particularly in the south with a total ban on hill tops and upper slopes where trees are due to be planted [9]. As an unintended consequence, the regenerated landscape may become favourable again to populations of small mammal intermediate hosts of *Echinococcus* spp. [13]. Rehabilitation of degraded areas has been shown to cause increased population densities of intermediate hosts (especially) and definitive hosts (dogs and foxes) for *Echinococcus* spp. [9]. Additionally, legislation banning indiscriminate use of rodenticides in NHAR from 2002 has further added to increases in the rodent and dog populations [8,13].

### *Echinococcus* spp. life-cycle in animals and transmission to humans

Definitive hosts of *E. granulosus* in NHAR are dogs and other canids while intermediate hosts include sheep, goats, swine, cattle, horses, camels and, according to our recent research findings, rodents [13] and other small mammals, such as lagomorphs [23]. *E. multilocularis* has a similar life cycle to *E. granulosus* with the main definitive hosts being foxes and dogs, and the intermediate hosts are small mammals especially rodents. Roaming domestic dogs can provide a transmission link between the sylvatic cycle and humans, thereby increasing the risk of human infection. It is known that dogs are generally highly susceptible to infection with *E. multilocularis*[24]. Free-roaming/scavenging behavior leads to infection, especially in under-developed southern NHAR where dogs are poorly fed and there is a large population of small mammals in close proximity to households. Humans become accidentally infected by ingesting *Echinococcus* eggs from any of three sources: an egg-contaminated natural environment; ingestion of contaminated foodstuffs or water; or through direct contact (e.g. playing) with infected domestic dogs. After ingestion, the eggs release oncospheres in the intestine leading to the development of cysts in various organs [25], particularly the liver [6]. It can take up to 10-15 years for the symptoms of infection to manifest and be diagnosed [2].

#### Intermediate hosts

##### Rodents/small mammals

Historically, the first report from southern NHAR of the ground squirrel, *Spermophilus dauricus* and *S. alashanicus* infected with *E. multilocularis* was in 1985 [26,27]. Both *Spermophilus* spp. and *Myospalax fontanieri* were found to be infected with fertile AE cysts containing protoscoleces [28]. Other suitable intermediate hosts such as *Cricetulus* spp. and *Meriones unguiculatus* were recorded in scrub and grassland in the Liupan Mountains [29], though without parasitological examination. During the 1980s, prevalence of AE was 0.6% in *Citellus* spp, 0.2% in *Citellus dauricus,* and 0.3% in *Myospalax fontanierii*[26].

The relationship between landscape and small mammal population dynamics in regulating *E. multilocularis* transmission has been studied extensively. A strong environmental predictor of small mammal habitat suitability is the ratio of optimal to marginal patch area (ROMPA) [12]. Landscape changes can have a major effect on small mammal communities and may play a role modifying cestode transmission patterns involving small mammals. For example, deforestation may increase the area of habitats favourable for *Spermophilus dauricus/alashanicu,* also referred to as *S. dauricua or Myospalax fontanieri*[13]. This large rodent is one of the commonest rodent species in the southern NHAR area (accounting for around 80-85% of all rodent species) [18], largely occurring in fields, fallow areas and in areas with early re-forestation [8,13]. It was also recently found to be susceptible to natural, patent infection with the larvae of *E. granulosus*[13]. Despite providing no definitive proof of a cycle involving ground squirrels and dogs/foxes, this finding highlights the possibility for high transmission intensity due to heavy environmental contamination with *E. granulosus* eggs [8,13,30] in this region of NHAR. The study also indicated that rodents can be shared intermediate hosts for *E. multilocularis* and *E. granulosus*[13], and that they may serve as valuable indicators for assessing the occurrence and the level of environmental contamination with eggs of these parasites and the infection pressure resulting.

##### Livestock

Families in the southern rural areas of NHAR, usually raise sheep and cattle, and keep dogs to help manage their livestock. When necessary, livestock are slaughtered by the owners, close to their dwellings and often near a river bank, to facilitate the washing of carcasses. Owners report feeding raw viscera (sometimes known to have hydatid cysts) to their dogs or they discard the viscera, especially if they contain cysts. It is likely that free-roaming dogs become infected with *E. granulosus* after feeding on discarded sheep offal containing larval *E. granulosus*. An investigation in the mid-1980s found the prevalence of CE in sheep in Xiji, Haiyuan and Guyuan (XHG) counties to be 79% and 40% in Tongxin County [18]. In the same study, the prevalence in goats in Tongxin was 21% [18]. Up until the late 1990s, livestock records from local abattoirs showed a high prevalence of CE in sheep (52%), cattle (81%), pigs (24%) camels (19%) and goats (3%), in the hyper-endemic counties of NHAR [31].

Re-forestation of eroded landscapes started in NHAR from the beginning of the 1990s, limiting the land available for sheep grazing. Sheep and other livestock are raised generally in enclosed yards, and the numbers of livestock kept by families have been reduced [8]. CE is still found regularly in sheep and other livestock in abattoirs although recent reports suggest that the occurrence of infected animals at slaughter is lower than 20 years ago [32]. Infection in sheep usually occurs on farms where domestic dog management is poor and/or where there is a problem with large populations of roaming dogs. Infected dogs will contaminate the pasture with eggs of *E. granulosus,* which sheep ingest while grazing. According to earlier reports, *E. multilocularis* can be found under natural conditions in various mammalian hosts (e.g. horses, domestic and wild pigs) but they do not play a role in the transmission cycle [33]. As far as we are aware, there have been no reports of larval *E. multilocularis* infection in Chinese livestock. Suspected *E. multilocularis* infection in Tibetan yaks was found to be misdiagnosed [34].

### Definitive hosts

#### Dogs and foxes

In the mid-1980s, Li and colleagues were the first to report *E. multilocularis* infection in foxes in NHAR, finding 3/20 (15%) trapped red foxes from Xiji and Guyuan Counties to be infected [18,27]. The infection intensity varied from 1,840–4,050 tapeworms. This investigation also found 4/385 (1%) domestic dogs in Xiji County infected with *E. granulosus* by necropsy. Veterinary records of necropsies from Tongxin County in 1991-92 indicated an infection prevalence of over 55% in dogs [31]. However, in the two decades since then, there has been a lack of veterinary surveillance data, possibly for two reasons: 1) the government veterinary service in NHAR has received poor financial support from the mid-1990s, since restructuring of the economic system [17], resulting in a lack of trained staff; and 2) the dog populations dramatically declined due to the introduction in the early 1990s of rodenticides (used in poisoned baits) to control the large rodent population present, resulting in inadvertent poisoning of a large number of dogs. As we described in a previous report [9], intensive deforestation and increased ploughing of fields resulted in an increase in the local rodent population, and a reduction in the numbers of predators (particularly foxes) due to the loss of habitat. When we undertook community surveys at the beginning of the 2000s, dogs were completely absent in some villages in southern rural NHAR, or only rarely in others [21], where dogs used to be numerous [18,22]. In contrast, in urban areas of southern NHAR, the population of dogs, which are generally well-fed, remained constant. From 2002, the dog population in rural villages grew dramatically because of the local government regulations prohibiting the use of anticoagulant drugs (used as rodenticides) and their sale in markets [8]. According to veterinary reports, the dog population (including stray and owned dogs) is currently similar in number to the human population in some counties of southern NHAR [32]. This change may lead to increased risk of human AE or CE if it is coupled with increased densities of small mammal species susceptible to the *Echinococcus* spp., due to the environmental changes occurring, and a failure to prevent home slaughter of livestock animals and poor management of raw viscera.

### Social and behavioural factors influencing transmission

Rural villagers in NHAR engage daily in the collection of herbs and wild vegetables and hunting of wild animals, especially large mammals. Sheep farming is also a major economic activity for these rural communities, especially those dominated by the Hui ethnic group (Figure 2). Because there has been almost a complete lack of piped water in past decades, these people have relied mainly on natural sources such as seasonal rivulets, rain collection and temporary wells dug in dry-river beds for their water supply. Livestock and domestic dogs, together with wild animals, are allowed free access to these water supplies, leading to contamination with animal faeces, thereby increasing the risk of the transmission of *Echinococcus* spp.[13]. Indeed, our recent human surveys in NHAR revealed considerable numbers of patients with CE and AE [21], reflecting a high level of *Echinococcus* transmission over previous decades since the incubation periods of human CE and AE are generally very long (about 10-15 years).

Farming households in rural NHAR typically have a well (often uncovered), in addition to stables and pens for livestock, dog kennels and latrines within the vicinity of the dweling. On a daily basis, human faeces (‘night-soil’) and livestock and dog faeces are mixed with soil to create compost for use as fertiliser. The compost is generally stored in a corner of the yard, a practice that may not only increase *Echinococcus* egg survival time if any are present in dog faeces, but which also increases the chance of contaminating the local water supply (e.g. nearby wells) with ova when heavy rainfall occurs. Additionally, water for consumption is usually not boiled by these rural communities [21].

### Changes in human echinococcosis prevalence/incidence over time

Up until the late 1980s, 304 human AE cases had been diagnosed from the hyperendemic southern counties of NHAR [19]. A mass-screening survey conducted in communities in Xiji County in 1989 [35] indicated that in two townships (Xinying and Baiya), 8.2% of subjects had echinococcosis, including 5.9% with AE and 2.3% with CE. Diagnosis was by ultrasound and serology and subjects were aged 19–72 years [35]. Another community survey using ultrasound in Yumu village in 1996 showed a human AE and CE prevalence of 4.6% [19]. A total of 113 clinical AE cases were diagnosed in Guyuan hospital between 1965 and 1991 [36]. Two reports had described family clusters of cases [22,37]; these families hunted ground squirrels (*Spermophilus* spp. for meat and gave the raw viscera to their dogs [22]. Previous incidence values recorded for human echinococcosis in other parts of China [37] and in NHAR [20] were under-estimates, because the hospital records would have excluded individuals who were asymptomatic, or who had not sought clinical advice due to their poor economic status. Therefore, active surveys, undertaken (2001-2003) in communities in Xiji, Guyuan and Longde counties, showed a heterogeneous prevalence between communities, ranging from 0–8% (mean 2%) for AE and 0–7.4% (mean 1.6%) for CE, although the overall prevalence was lower (3.6%) than in previous reports [21]. The variable prevalence of human AE and CE among communities may be associated with different micro-environmental features of the localities.

The co-existence of both AE and CE in most communities within southern NHAR indicates that the environment was conducive to the transmission of both forms of the disease. Although human CE cases have been found throughout the whole of NHAR, the absence of AE in the north may be attributed to the hot and dry climate and other risk factors providing unsuitable conditions for *E. multilocularis* egg survival [38]. Clinical records (1992-2002) for the whole of NHAR showed an age range of cases of 1–80 years for CE, but 21–70 years for AE [6]. By contrast, community surveys [21], only conducted in southern parts (Xiji, Guyuan and Longde counties) of NHAR in 2002-2003, found an age range of 19–79 for both AE and CE. This indicates that there were very different spatial patterns of transmission in the intervening period. The age distribution of patients (many older-aged patients and few younger-aged patients) provides indirect evidence that AE and CE transmission had been very high some decades ago but had been reduced (even stopped) by the collapse in definitive host (dogs/fox) populations in southern NHAR due to anthropogenic environmental change and the changes in policy described earlier [8,9]. The relative youth of some CE patients (identified using hospital records) indicated CE transmission was still very active in other areas of NHAR compared with the south [6].

While clinical echinococcosis was found to be very rare in young people in a recent investigation [39], sero-prevalence amongst this group showed an increased trend in many southern NHAR communities [21,30]. Exposure to *Echinococcus* can be diagnosed after only a few months by identifying specific antibodies in human serum [40]. The increase in sero-prevalence among younger subjects is of concern because it suggests that the incidence of clinical cases is likely to rebound in NHAR, with an increase in the burden of echinococcosis in coming decades.

### Changes in the transmission patterns of echinococcosis over time

The micro-climate is important for the survival rates of *Echinococcus* eggs once they have been shed into the environment [9,41]. The higher concentration of infectious definitive host faeces can lead to parasite over-dispersion, which, for example, can be caused by eggs spreading widely, due to rain or washing, or the use of faeces as fertiliser. Additionally, the burying of faeces (e.g. by cattle compacting and turning over soil, by the deposition of eggs into muddy soil near water courses or by humans mixing manure and soil to form compost) may increase parasite egg survival rates [7], thus increasing the likelihood of their ingestion by intermediate hosts and humans. Furthermore, the behaviour of local sub-groups of foxes or stray dogs around a village may be more important than the common behaviour of the total canine host population for human infection [10].

The landscape composition and structure of micro-areas is also important for the maintenance of small mammal and fox populations, thereby ensuring the completion of a wildlife cycle for *E. multilocularis*. Landscapes with pasture and grassland are also important for the provision of grazing for sheep and other livestock, which are necessary to maintain a domestic cycle for *E. granulosus.* The impact of these landscape and micro-environmental factors was emphasised by a recent series of studies which found evidence of hot-spots of transmission and geographical clusters of human AE and CE cases [10,12,16] that have changed over time. Clear evidence of this is provided by the heterogeneous distribution we described earlier of human AE and CE cases in various communities of southern NHAR [21]. Furthermore, a previous community survey that we undertook in a hyper-endemic area in NHAR (Xiji County) using filter paper blood-enzyme-linked immunosorbent assay (ELISA) for human antibody detection against *E. multilocularis* protosocolex antigen (anti-EmP) - which measures *E. multilocularis* egg exposure - revealed that sero-prevalence significantly increased in children in some village communities (Huoshizhai and Nanwan), whereas in others (e.g. Haoziwan), the sero-prevalence, detected by ELISA against *E. granulosus* hydatid cyst fluid lipoprotein antigen (anti-EgB) - which indicates *E. granulosus* egg exposure - had decreased [30] (Table 1). This combined evidence illustrates the fact that transmission of the *Echinococcus* spp. has changed spatiotemporally, together with simultaneous environmental and climatic changes in rural southern NHAR [30]. Similar findings have been reported from other echinococcosis-endemic areas worldwide [11].

**Table 1:** **The presence (+) or absence (-) of sero-positive teenagers and ultrasound-detected adult cases of cystic and alveolar echinococcosis in various village communities in hyper-endemic Xiji County, People's Republic of China from 2001-3**[21,30]

	**Ultrasound detected cases**		**Sero-positive subjects**	
Age range	20-79 yrs old		6-18 yrs old	
Disease^a^	AE	CE	Anti-Em^b^	Anti-Eg^c^
Huoshizhai^d^	+	+	+	-
Nanwan	+	+	+	-
Haoziwan	+	+	-	+
Zhangcunpu	+	+	+	+
Bai’ai	+	+	+	+
Baicheng	-	+	+	+

^a^AE, alveolar echinococcosis; CE, cystic echinococcosis.

^b^Anti-Em, exposure to *E. multilocularis* eggs measured by anti-EmP ELISA.

^c^Anti-Eg, exposure to *E. granulosus* eggs measured by anti-EgB ELISA.

^d^Name of village community.

### Potential spread of *Echinococcus*

The Chinese government has changed its policy to re-forestation of eroded landscapes in NHAR. As a result, landscapes there may become favourable again for *Echinococcus* spp. transmission when the intermediate stages of forest succession become widespread and the improved environmental conditions provide suitable wildlife habitats for rodents and foxes and increased grazing resources for livestock. If these circumstances are coupled with immigration of *Echinococcus*-infected definitive hosts, this will allow the parasites to disperse within these new areas. Currently, there is an increasing market throughout China for guard dogs [9]. If there is no regulatory system and limited inspections to monitor diseases in dogs, this trade could contribute to the dispersal and transmission of *Echinococcus* spp. within China. Dogs will present a major risk for spreading these parasites to non-endemic areas not only in NHAR but elsewhere in China, such as the Tibetan plateau, where the situation for transmission may be comparable.

### The implementation of control measures for echinococcosis in NHAR and the challenge

The transmission and the risk of *Echinococcus* infection can increase as the result of anthropogenic and natural environmental changes in NHAR, but control, if implemented effectively, can limit the effect. The national echinococcosis control program (NECP) for China, which was supported and launched by the Chinese Government in 8 provinces/autonomous regions from 2005 [3], has been in place in NHAR since 2006. The main activities of the NECP, which include canine purgation and praziquantel treatment of registered dogs, culling of unwanted and stray dogs, health education, and improved slaughter house management and inspection of livestock carcasses, have already impacted favourably on echinococcosis control in NHAR [42]. A major challenge to control of echinococcosis in NHAR is the very large numbers of stray dogs, not only in rural areas but also in cities [43], which increases the likelihood of continual transmission.

### Conclusions

It is very important to understand the natural history of the transmission cycles of echinococcosis in NHAR in order to quantify the impact of the 20–30 year process of deforestation, agricultural expansion and land use changes on the disease. These anthropogenic acitivities have led to dramatic natural environment changes and ecological diversities of the hosts of *Echinococcus* spp., consequently affecting parasite transmission between animal and human hosts. It is vital that such information, based on detailed and continually updated epidemiological data, is obtained to assist in the design of cost-effective public health programs to control echinococcosis in NHAR and other areas of China endemic for the disease.

## Competing interests

The authors declare that they have no competing interests.

## Authors’ contributions

YRY and DPM conceived the idea for the review. YRY prepared the first draft of the manuscript. DPM, ACAC, DJG, J-AA, GMW and TSB provided critical comments and helped in drafting subsequent revisions. DPM finalised the manuscript. All authors read and approved the final manuscript.
